# The Complex Roles of Adipokines in Polycystic Ovary Syndrome and Endometriosis

**DOI:** 10.3390/biomedicines10102503

**Published:** 2022-10-07

**Authors:** Susanne Schüler-Toprak, Olaf Ortmann, Christa Buechler, Oliver Treeck

**Affiliations:** 1 Department of Gynecology and Obstetrics, University Medical Center Regensburg, 93053 Regensburg, Germany; 2 Department of Internal Medicine I, University Medical Center Regensburg, 93053 Regensburg, Germany

**Keywords:** polycystic ovary syndrome, endometriosis, adipokine, batokine, white adipose tissue, brown adipose tissue, obesity, infertility, single nucleotide polymorphism, omics

## Abstract

Polycystic ovary syndrome (PCOS) and endometriosis are frequent diseases of the female reproductive tract causing high morbidity as they can significantly affect fertility and quality of life. Adipokines are pleiotropic signaling molecules secreted by white or brown adipose tissues with a central role in energy metabolism. More recently, their involvement in PCOS and endometriosis has been demonstrated. In this review article, we provide an update on the role of adipokines in both diseases and summarize previous findings. We also address the results of multi-omics approaches in adipokine research to examine the role of single nucleotide polymorphisms (SNPs) in genes coding for adipokines and their receptors, the secretome of adipocytes and to identify epigenetic alterations of adipokine genes that might be conferred from mother to child. Finally, we address novel data on the role of brown adipose tissue (BAT), which seems to have notable effects on PCOS. For this review, original research articles on adipokine actions in PCOS and endometriosis are considered, which are listed in the PubMed database.

## 1. Obesity and Adipokines

The prevalence of obesity continues to spread globally [1]. Excess adipose tissue mass and adipose tissue dysfunction develop in obese individuals and affect almost all physiological processes, since adipose tissue has been recognized as a major endocrine organ acting by the secretion of specific signaling proteins: the adipokines [2]. Adipose tissue can be classified into white adipose tissue (WAT) and brown adipose tissue (BAT), which secrete different sets of adipokines. WAT consist of large adipocytes with a single lipid droplet and few mitochondria and thus has the capacity for energy storage and homeostasis in response to nutritional demands. In contrast, BAT is comprised of adipocytes with smaller lipid droplets but with a high number of mitochondria resulting in its ability to produce heat [3]. The effects of obesity on health are strongly dependent on the location of WAT within the body [4]. In humans, according to its distribution, WAT is classified in two main depots: visceral WAT (VAT), comprising of omental and mesenteric WAT, which is known to trigger adverse metabolic effects, and subcutaneous WAT (SAT) [5]. High amounts of VAT are associated with insulin resistance and glucose intolerance as a result of metabolic deterioration [6,7,8]. Consequently, the size of the VAT depot is directly associated with a poor prognosis in metabolic diseases, and a high VAT mass has been shown to be an independent factor for metabolic dysregulation [9]. In contrast, SAT has been proposed to exert protective roles, as it is associated with glucose tolerance, insulin sensitivity and a low risk for type 2 diabetes after adjustment for BMI [5,8].

Brown adipose tissue (BAT) is functionally and structurally different from white fat tissues. In contrast to white adipocytes, brown fat cells have multilocular lipid droplets. BAT is rich in mitochondria and thus has an important adaptive thermogenic capacity for cold defense via uncoupling protein 1 (UCP1). BAT thermogenesis functions to turn excess energy into heat to maintain the homeostatic energy balance [10]. Mechanistically, cold exposure causes the release of norepinephrine, which binds to the BAT-expressed beta 3 adrenergic receptors, activating heat production in BAT [11]. The thermogenic activity of BAT is related to metabolic health and is impaired in obese individuals [12]. Body mass index (BMI) and age are inversely correlated with BAT activation [13]. BAT content declines in the obese and the brown fat cells acquire a white fat cell-like morphology [14]. Thus, BAT has become a promising target for the therapy of metabolic diseases [15]. BAT secretes various metabolites, such as lipids and proteins (brown adipokines), which are collectively called batokines. A comparison of the secretome of human white and brown fat cells identified 101 proteins that were specifically released from BAT [16]. Fibroblast growth factor 21 (FGF-21) is a brown adipokine secreted from BAT upon thermogenic activation. FGF-21 is expressed by many different cells and affects the ovaries, brain, brown and white fat tissues as well as skeletal muscles and the heart. Its role is to regulate the bodies’ response to nutritional stress [17]. IL-6, myostatin, neuregulin 4 and growth-and-differentiation factor 15 are batokines regulating the function of various organs, such as the liver or skeletal muscle, and are also involved in disorders of the female reproductive system [18,19].

The function of adipose tissue as a highly active endocrine organ emerged with the discovery of adipokine leptin nearly 30 years ago [20]. Leptin is primarily produced by WAT adipocytes and small-intestine enterocytes and its main function is to regulate energy balance by inhibiting hunger, which in turn diminishes fat storage in adipocytes. Leptin regulates adipose-tissue mass through hypothalamus effects on hunger and energy use. In hypothalamic neurons, an adequate leptin-receptor function and the subsequent regulation of energy metabolism and body weight depends on interactions of the receptor with gangliosides in the cell membrane [21]. Leptin has been suggested to be a very good biomarker of adiposity, as its circulating levels are in linear proportion to fat mass [22]. The reduction in circulating serum leptin activates processes to increase body energy reserves and suppresses energy-demanding functions, such as reproduction [23]. In obese humans, systemic leptin is increased, but the anorexigenic efficacy of this adipokine is strongly impaired [24]. Leptin signals through the different leptin-receptor (LEP-R) isoforms, and loss of leptin as well as the leptin-receptor function, has been linked to extreme obesity, immune dysfunction and infertility [25]. The quantity of functionally active LEP-R is affected by a constitutive shedding of the extracellular domain. The product of this cleavage process, the so-called soluble leptin receptor (sLEP-R), is the main binding protein for leptin in human blood and thus modulates its bioavailability. Concentrations of sLEP-R are differentially regulated in metabolic disorders, such as type-1 diabetes mellitus or obesity, and can, therefore, enhance or reduce leptin sensitivity [26]. Both leptin and its membrane and soluble receptors play an important role in polycystic ovary syndrome (PCOS) and female reproduction [27,28].

In contrast to most other adipokines, serum adiponectin declines in obese individuals [29]. The very-high plasma concentration (about 1000-fold higher than that of other adipokines) is unique to adiponectin [30]. Adiponectin, mainly secreted from WAT but also from BAT, is a very potent insulin sensitizer and protects from type-2 diabetes and atherosclerosis. This adipokine increases energy expenditure, enhances the uptake of glucose by skeletal muscle cells and adipocytes, stimulates fatty acid oxidation and decreases hepatic gluconeogenesis [25]. Higher-order multimers are formed in adipocytes and there is strong evidence that the metabolic activities of adiponectin are mostly exerted by these forms [31]. Adiponectin receptors 1 and 2 are the main adiponectin receptors in vivo, and recently T-cadherin was identified as another adiponectin receptor [32,33]. Numerous adiponectin-receptor agonists showed promising results in preclinical experiments and most likely will be tested in clinical studies soon [34]. Adiponectin has been reported to play a role in diseases of the female reproductive tract, as is elaborated on below [35,36].

Omentin-1 (also known as intelectin-1) is another adipokine whose systemic levels decline in obese individuals. This adipokine is highly expressed in VAT and hardly detectable in SAT [37]. Omentin-1 protects from type-2 diabetes, atherosclerosis and cancer. This protein reduces oxidative stress, inflammation and programmed cell death [38]. Omentin-1 receptors, αvβ3 and αvβ5 integrins, have been identified in macrophages [39]. The plasma levels of this adipokine are decreased in women with PCOS, suggesting a role of omentin-1 in this disease [40].

Adipokine chemerin is secreted from WAT, and also from hepatocytes [18,41]. Consistent recent proteomic studies have identified chemerin to also be secreted from activated BAT [18,42,43,44]. Chemerin expression in BAT is induced in obese individuals, which might result from the observed BAT whitening in obese individuals [45]. Chemerin acts, context dependently, as a pro as well as an anti-inflammatory protein [46]. Chemerin was shown to regulate adipogenesis, glucose metabolism and blood pressure [45,47,48]. Chemokine-like receptor 1 (CMKLR1) and G-protein-coupled receptor 1 (GPR1) mediate chemerin signaling. C-C chemokine receptor-like 2 (CCRL2) binds chemerin and increases its local concentration [49,50]. Chemerin is a chemotactic protein for immune cells, and a chemerin gradient induces leukocyte migration [51]. Serum chemerin is increased in obesity, but there is a disparity between active and total chemerin levels [52]. Chemerin has been reported to play a role in PCOS [53].

Visceral adipose tissue-derived serine protease inhibitor (vaspin) is another adipokine, which protects from inflammation, liver steatosis, atherosclerosis and insulin resistance. In the fat of lean humans, vaspin mRNA was not detectable and its expression was induced in the VAT of obese individuals [54]. However, the observation of a predominant expression of vaspin in visceral fat has been challenged, resulting in the detection of high vaspin expression in SAT [55]. Vaspin is a member of the serine protease-inhibitor family and was classified as serpin A12 [56]. Kallikrein 7 is a protease target of vaspin and the inhibition of kallikrein 7 by vaspin improved glucose metabolism by the elevation of insulin plasma concentrations [57]. Vaspin can also exert effects through the cell-surface 78-kDa glucose-regulated protein (GRP78) in complex with the DnaJ-like protein 1 [58]. Serum vaspin levels were increased in the obese and type-2 diabetic patients compared with the healthy controls [59,60].

Apelin is a relatively short protein of 77 amino acids and can be cleaved to different active isoforms, such as apelins-13 and -17. All apelin variants can bind to the G-protein-coupled apelin receptor APLNR [61]. Apelin and APLNR are widely expressed and were also detected in WAT adipocytes [61,62]. Apelin improves glucose tolerance, insulin sensitivity, glucose uptake of skeletal muscle and adipocytes, biogenesis of mitochondria and fatty acid oxidation in skeletal muscle cells. Apelin protects from obesity, hypertension, glucose intolerance and insulin resistance [25]. Moreover, the antifibrotic activities of apelin have been described. On the other hand, apelin was also shown to contribute to inflammation and tissue fibrosis [63,64]. Serum apelin was increased in obese patients, Ref. [61], with types 1 or 2 diabetes [65,66]. Serum levels of apelin-17 but not apelins-36, 13 or 12 were reported to be higher in obese women with hypertension than in those with normal blood pressure [67].

Visfatin (nicotinamide phosphoribosyltransferase, pre-B-cell colony-enhancing factor) generates the nicotinamide mononucleotide for nicotinamide adenine dinucleotide (NAD) biosynthesis [68]. NAD-consuming enzymes regulate various cellular processes indicating an essential role of intracellular visfatin. VAT and SAT tissues express comparable levels of visfatin [69]. Secreted visfatin was shown to exert inflammatory and profibrotic activities [70,71,72]. Notably, visfatin up-regulated fibrotic proteins in adipocytes [73]. Adipose tissue fibrosis is related to insulin resistance, liver steatosis and metabolic diseases [74]. Visfatin expression is elevated in the adipose tissues of obese patients, and its serum levels are increased in obesity [75]. A meta-analysis conducted on 13 studies showed that plasma visfatin levels were higher in overweight/obesity, type-2 diabetes mellitus, metabolic syndrome and cardiovascular diseases [76].

Follistatin, originally identified in follicular fluid capable of inhibiting the follicle-stimulating hormone (FSH), is expressed in several tissues, including the ovary, pituitary, muscle and stromal vascular cells in WAT [77]. This protein functions as an antagonist for activin A, which inhibits adipogenesis. The inhibitory effect of follistatin on activin A and additional members of the transforming growth factor (TGF)-β superfamily, such as myostatin and bone morphogenetic proteins, was shown to protect from metabolic diseases in experimental models [78]. Circulating follistatin is not changed in obesity [79]. Notably, follistatin mRNA is reduced in the SAT of obese woman and normalizes upon weight loss [77]. Follistatin is involved in many disorders of the female reproductive system [79,80]. In Table 1, the main functions of the indicated adipokines are presented.

Adipokines affect gynecological disorders and female fertility [81]. Generally, fertility in women is a subtly balanced arrangement involving anatomy, hormones, environment, genetics, the immune system and many other factors [82]. Minor defects in the single parts of this construct lead to considerable consequences. Two gynecological disorders that are known to result in infertility are polycystic ovary syndrome (PCOS) and endometriosis [83,84]. Both diseases have distinct characteristics. However, among others, the induction of chronic inflammation and activation of proinflammatory pathways are two molecular mechanisms they have in common. In the following sections, the role of adipokines in these disorders is addressed.

## 2. Polycystic Ovary Syndrome

Polycystic ovary syndrome (PCOS) is a widespread endocrine disorder among women of a reproductive age, with a prevalence of up to 15% [85]. Notably, the increasing incidence of obesity in the recent decades was accompanied by an elevated prevalence of PCOS. Despite its high prevalence and economic burden, the pathogenesis of PCOS is still unclear. Although the heritability of PCOS is estimated at 70%, to date the genetic loci linked to this disease account for only ~10%. Recent studies suggested that epigenetic changes resulting from a hormonal dysregulation of the maternal uterine environment are further mechanisms involved in the etiology of PCOS [86]. PCOS is a heterogeneous disease characterized by the presence of at least two of the following conditions: hyperandrogenism, ovulatory dysfunction and polycystic ovarian morphology, often resulting in infertility [87,88]. This syndrome is associated with hyperinsulinemia, insulin resistance, hyperlipidemia and chronic low-grade inflammation, often leading to the development of metabolic syndrome and cardiovascular diseases [89,90,91,92]. Its pathogenesis, including heredity, epigenetic mechanisms, environment, living habits and diet, is complex. During the prenatal period, not only genetic and epigenetic factors but also an excess amount of anti-Mullerian hormone (AMH) and androgen, growth restriction and endocrine disruptors, such as bisphenol A (BPA), predispose women to the development of a PCOS-like phenotype in adulthood [93]. Androgen excess, one of the disease-characterizing conditions, is suggested to play a prominent role in the development of metabolic disorders affecting different types of tissues, such as adipose tissues, the liver, pancreas, muscle and brain [94]. The question of why hyperandrogenism is associated with PCOS in nearly 80% of the cases has not been completely understood to date. An intrinsic abnormality in the steroidogenic machinery of the ovarian theca cells is suggested to be one reason for increased androgen biosynthesis frequently associated with PCOS [94,95].

The diagnostic criteria for PCOS subgroups have evolved in recent decades, but global consensus was not achieved [96]. The introduction of the Rotterdam criteria in 2003 led to a substantial increase in the number of patients diagnosed with PCOS, and broadened the heterogeneity of PCOS phenotypes as compared with the National Institutes of Health’s (MIH) definition from 1990 [97]. The Rotterdam criteria contain oligo- or anovulations, clinical and/or biochemical signs of hyperandrogenism and polycystic ovaries. Two out of these three criteria have to be present for the diagnosis of PCOS. Moreover, other etiologies, such as congenital adrenal hyperplasia, androgen-secreting tumors or Cushing’s syndrome, have to be ruled out [87]. However, NIH’s 2012 phenotypic extension of the Rotterdam definition, defining four PCOS subtypes (A-D), has been suggested to be the most convenient approach when conducting research and clinical practice. This approach permits comparisons in epidemiologic studies among different populations and allows researchers to identify high-risk individuals in clinical practice. These are phenotype A, comprising hyperandrogenism, ovulatory dysfunction and polycystic ovarian morphology (PCOM); phenotype B, comprising hyperandrogenism and ovulatory dysfunction only; phenotype C, comprising hyperandrogenism and PCOM; and finally phenotype D, comprising ovulatory dysfunction and PCOM [98]. Recently, biochemical and genotype data obtained from a PCOS genome-wide association study (GWAS) led to the postulation of only two subtypes of PCOS, a “reproductive” type (21–23% of all cases), characterized by low BMI, glucose and insulin levels, higher luteinizing hormone (LH) and sex hormone-binding globulin (SHBG), and a “metabolic” group (37–39%), characterized by higher BMI, glucose and insulin levels, but lower SHBG and LH levels [99]. The difficulties to achieve a global consensus on PCOS criteria, however, clearly show and emerge from the heterogeneity of PCOS. Further attempts employing all methods of contemporary omics are needed to fully elucidate the pathogenesis of this disease. Given that PCOS is a disease with strong evidence of a genetic component, since the advent of GWAS, genetic studies of PCOS have progressed considerably. Single nucleotide polymorphisms (SNPs) in genes involved in insulin, cytokine, vitamin-D, hormone and adipokine signaling have been identified as PCOS-susceptibility markers [100]. Furthermore, epigenetic modifications in the pathogenesis of PCOS have come into the focus of interest, and changes in DNA methylation have been observed in the serum, ovaries, hypothalamus, skeletal muscle and adipose tissue of PCOS patients [101,102]. Transcriptomics allow the identification of aberrantly expressed genes in the granulosa or cumulus cells of women with PCOS and have already revealed genes with altered expressions, including non-coding RNAs, such as miRNAs and lncRNAs, which might contribute to ovarian dysfunction [103,104]. Finally, proteomics are used, e.g., to identify novel protein biomarkers in the serum or follicular fluid (FF) of PCOS patients.

### 2.1. Role of Adipokines in Polycystic Ovary Syndrome (PCOS)

Adipokines affect metabolic and endocrine signaling in women with PCOS. Several adipokines are known to affect the regulation of the hypothalamic–pituitary–gonadal axis or to locally alter ovarian steroidogenesis. In women with PCOS, the dysregulation of adipocyte-secreted adipokines has been reported, e.g., these women have lower serum levels of adiponectin and higher leptin levels.

The latest research using different omics approaches revealed SNPs in adipokines and their receptors as PCOS-susceptibility markers, and epigenetic changes conferred from mother to child modulating adipokine expression, increasingly becoming a focus of interest, have already substantiated the importance of adipokines in PCOS [100,105]. However, the majority of classical studies addressing the role of adipokines in PCOS examined levels of circulating adipokines [106]. Further studies examined adipokine levels in follicular fluid (FF) or their gene expression in the ovaries of PCOS patients [107,108,109]. In vitro studies were performed to elucidate the molecular mechanisms underlying adipokine action in this disease [110]. Furthermore, the role of BAT in PCOS pathogenesis and therapy has attracted much attention lately [111]. Furthermore, considering all these aspects, the role of WAT adipokines, but also of BAT and some of its batokines in PCOS, including the latest studies, was primarily addressed in this study. In Table 2, we first present an overview of the main functions of the indicated WAT adipokines in PCOS patients.

#### 2.1.1. Leptin in PCOS

Since the identification of adipokine leptin about 30 years ago, a multitude of studies addressed the role of leptin in PCOS by comparing serum levels of this adipokine in women with and without PCOS, in the majority considering general parameters, such as obesity, and PCOS-associated parameters, such as hyperandrogenism. However, since then, a notable number of conflicting reports on this topic have been published. Considering the recent studies only, a clear trend towards an observation of elevated leptin serum levels in PCOS patients is emerging. A meta-analysis conducted on 19 studies, including 991 women with PCOS and 898 controls, revealed a small but statistically significant increase in leptin serum levels in the PCOS group [112]. This observation was further corroborated by the latest, although relatively small studies. A study from 2022, including 89 PCOS patients and 139 healthy women, reported leptin serum levels to be substantially higher in women with PCOS than in the control group (15.20 ng/mL vs. 9.71 ng/mL, respectively) (*p* < 0.001). Elevated leptin serum levels were present both in the lean and obese subgroups of PCOS patients [27]. In contrast, in another recent study, including 224 PCOS patients and 198 controls, elevated leptin serum levels were reported to be present in lean PCOS patients only [113]. Significantly elevated leptin serum levels in women with PCOS (*p* < 0.001) were also reported in a recent, albeit small study, each including 45 PCOS patients and controls [114]. Since the identification of the soluble form of leptin receptor (sOB-R or sLEPR), studies on its serum concentration in women with PCOS emerged. In a study including 122 PCOS patients and 81 controls, lean PCOS patients had significantly decreased sOB-R levels (*p* < 0.0001) compared to BMI-matched controls. Taking into account that low sOB-R levels supposedly compensate diminished leptin action, it was suggested that PCOS per se might cause leptin resistance [115]. The results of this study are supported by a later report, also observing decreased sOB-R levels in women with PCOS (in all patients and in the lean subgroup) [116]. Both studies suggested sOB-R to be involved in the pathophysiology of PCOS.

Several important studies elucidated the molecular mechanisms underlying the role of leptin in PCOS. Leptin was reported to modulate the reproductive function by regulating the central gonadotropin releasing hormone (GnRH) secretion, thus playing a role in the initiation of puberty and the periodic secretion of GnRH [117]. Increased leptin in follicular fluid was observed to be a sensitive marker of anovulatory fertility, a major problem of PCOS patients of a reproductive age [118]. Gene expression and circulating leptin levels were reported to be increased by insulin; thus, the insulin resistance in PCOS patients is suggested to elevate leptin secretion from WAT [119]. Leptin was recently reported to elevate the expression of aromatase genes in granulosa cells via mitogen-activated protein kinase (MAPK) and phosphoinositide-3 kinase (PI3K), whereas this effect was significantly reduced in the granulosa cells of PCOS patients [120]. Leptin was also shown to directly impair the insulin-like growth factor 1 (IGF-1)-mediated augmentation of FSH-stimulated E2 synthesis by granulosa cells, suggesting that high leptin levels may contribute to infertility in some women with PCOS by counteracting the sensitizing effects of IGF-1 in dominant follicles [121]. Taken together, leptin affects the occurrence and development of PCOS by regulating the reproductive endocrine axis and ovarian steroid production, and by participating in insulin resistance [122].

The pathogenesis of PCOS is considered to be determined by genetic, epigenetic and environmental factors. With regard to epigenetics, dysregulated metabolites in the bloodstream of the mother are thought to result in an epigenetic response to present an optimal environment for the developing fetus. The resulting epigenetic modifications can modify the expression of genes coding for metabolic receptors present in both the placenta and fetus. This process, which is called epigenetic sensing, is well studied, particularly for the insulin and leptin receptors. Maternal obesity alters the levels of these receptors or its ligands, for example, by epigenetic changes in the promoter region of these genes, as was reported for the leptin gene in the placenta [123], which may become permanent [124]. By analyzing the blood samples of pregnant women, it was observed that the BMI prior to pregnancy was related to the hypomethylation of the leptin gene promoter [125]. Furthermore, it has been reported that an abnormally elevated maternal level of leptin is associated with the adiposity of the newborn [126,127].

Genetic polymorphisms in the genes coding for leptin (*LEP*) and the leptin receptor (*LEPR*), particularly SNPs, have been demonstrated to be associated with adult obesity, childhood obesity or type-2 diabetes (reviewed in [128]). With regard to PCOS, several studies reported SNPs in the *LEPR* gene to be risk factors for the development of this disorder. A first study including 229 PCOS patients and 150 controls reported a highly significant association of Gln223Arg SNP in the *LEPR* gene with the development of PCOS (*p* < 0.0001) [129]. A subsequent study on 326 PCOS patients and 283 control individuals reported homozygous genotypes of *LEPR* SNP Lys109Arg (rs1137100) to be associated with PCOS (*p* = 0.012) [130]. A recent meta-analysis conducted on 33 studies identified a significant association of *LEPR* rs1137101 polymorphism with the susceptibility to PCOS in a recessive genetic model (*p* = 0.002, OR 1.88, 95% CI 1.26–2.78) [131]. Furthermore, in a very recent study, *LEPR* SNP rs1137101 was observed to be strongly associated with risk for PCOS (OR = 2.08, 95% CI 1.41–3.07, *p* = 2.0 × 10^−4^) in a dominant model, including 242 PCOS patients and 238 controls [132]. In conclusion, several SNPs in the *LEPR* gene were demonstrated to be risk factors for PCOS, whereas, to date, no *LEP* SNP has been identified as correlating with PCOS.

In conclusion, leptin affects the occurrence and development of PCOS by regulating the reproductive endocrine axis, ovarian steroidogenesis and by participating in insulin resistance. On the genetic level, SNPs, particularly in the *LEPR* gene, are polymorphisms that contribute to the heredity of obesity and are risk factors for the development of PCOS; on the other hand, maternal obesity exerts adverse epigenetic effects, e.g., on the *LEP* gene, which can be transferred to the fetus and might contribute to the adiposity of the child and might later affect PCOS risk. However, further studies on the molecular level are needed to unravel the complex function of leptin in this disease.

#### 2.1.2. Chemerin in PCOS

Chemerin and its receptor CMKLR1 are expressed in human granulosa cells and were reported to exert a higher expression in GC cells of PCOS patients [133]. In PCOS, these cells are known to be insulin resistant, associated with local hyperinsulinemia resulting in elevated chemerin expression [134]. Chemerin in turn has been reported to increase insulin resistance (IR) in the GC of PCOS patients [135], leading to an adverse positive feedback loop between chemerin and IR. High levels of chemerin binding to CMKLR1-expressing blood monocytes were reported to trigger local ovarian inflammation, leading to the apoptosis of granulosa cells, follicular growth arrest and anovulatory infertility [136]. Chemerin also has been suggested to be involved in excessive autophagy present in the granulosa cells of PCOS patients, which is associated with chronic inflammation [137,138,139].

A large body of evidence indicates elevated serum levels of chemerin in PCOS patients, supporting the involvement of this adipokine in the pathogenesis of PCOS. A major, recent meta-analysis comparing chemerin serum levels in women with and without PCOS, including 77 case-control studies with 8239 participants, reported significantly elevated circulating chemerin levels in PCOS patients with standard mean differences (SMD) of 1.87 with a 95% CI from 1.35 to 2.40 (*p* < 0.001) [106]. A further meta-analysis including 22 studies with 2256 participants also reported a significant increase in serum chemerin levels of PCOS patients in comparison to women without PCOS (*p* < 0.001) [109]. A subgroup analysis revealed chemerin serum levels to be significantly higher in overweight/obese women with PCOS (BMI > 25 kg/m^2^) than in overweight/obese women without PCOS (*p* < 0.001). Another meta-analysis including 8 studies with 897 participants corroborated the elevation of serum chemerin levels in PCOS patients compared to the non-PCOS group (*p* < 0.001) [140]. In non-obese women, chemerin levels did not differ between the PCOS and the non-PCOS groups [109]. In contrast, another meta-analysis conducted on non-obese women with PCOS, including 5 studies with 414 participants, reported varying degrees of increased chemerin serum levels in non-obese PCOS patients compared to the non-obese control group, which reached statistical significance (*p* < 0.03) [141]. In conclusion, chemerin serum levels were demonstrated to be elevated in PCOS patients compared to the non-PCOS group, with this increase being most pronounced in obese PCOS patients, but significantly lower or absent in non-obese women.

In some studies, chemerin levels in the follicular fluid (FF) of PCOS patients were examined, for example, those included in the meta-analysis of 22 studies with 2256 participants mentioned above, showing significantly elevated chemerin levels in the FF of PCOS patients compared to the control group (*p* < 0.001) [109]. Increased chemerin levels in the FF of PCOS patients were corroborated by a smaller study not included in the meta-analysis mentioned above [142]. Chemerin levels were reported to be increased in FF, but not in the serum of lean women with PCOS compared to a BMI-matched non-PCOS control group. The results of this study also support the concept that in PCOS, hyperandrogenemia increases chemerin expression promoting the recruitment of CMKLR1+ monocytes, deregulating the immunological niche of ovaries [136]. The elevation of chemerin levels in FF by ovarian hyperandrogenism of PCOS patients was also shown in a study reporting the correlation of theca cell androgens with increased levels of chemerin and other adipokines in FF, suggesting the existence of a close relationship between these two hormonal systems, which appear deeply involved in ovarian physiology and PCOS physiopathology [143].

Finally, the expression of the *RARRES2* gene coding for chemerin and of receptor CMKLR1 was examined in the GC of PCOS patients. Human luteinized granulosa cells (hlGC) of PCOS patients were reported to exhibit elevated mRNA levels both of *RARRES2* and *CMKLR1* genes resulting in reduced progesterone production by these cells being typical for PCOS. The results of CMKLR1-inactivation experiments suggested that chemerin via CMKLR1 could be involved in the altered steroidogenesis observed in PCOS [133]. In a small study including 23 PCOS patients and 55 controls, in GC cells, mRNA levels of *RARRES2* were reported to be higher in obese women with PCOS than in BMI-matched women without PCOS, and were strongly associated with chemerin levels in FF and with BMI [107]. In contrast, *CMKLR1* mRNA expression was considerably higher in non-obese women, particularly in PCOS-GC, than in the GC of obese women, irrespective of pathological status, with nearly undetectable mRNA levels. *CMKLR1* expression was negatively correlated with BMI and *RARRES2* expression, the latter suggesting a negative feedback loop between chemerin and its receptor, which was proposed previously [144].

Taken together, chemerin is up-regulated by hyperinsulinemia often present in PCOS and in turn has been reported to worsen insulin resistance (IR) in PCOS patients, e.g., by affecting the glucose utilization of granulosa cells, leading to an adverse positive feedback loop between chemerin and IR. It is suggested to contribute to local ovarian inflammation by the recruitment of immune cells and participating in excess autophagy of PCOS GCs and was reported to activate GC apoptosis and follicular growth arrest leading to anovulatory infertility. Thus, chemerin seems to support PCOS pathobiology by various mechanisms.

#### 2.1.3. Adiponectin in PCOS

Adiponectin is secreted by WAT and, as recent consistent proteomic studies demonstrated, also by BAT [18,42,43]. It increases fat metabolism, regulates glucose tolerance, maintains insulin sensitivity protecting individuals from type-2 diabetes (T2D) and displays anti-inflammatory activity in macrophages [145,146,147,148]. Adiponectin is suggested to counteract insulin resistance by boosting lipid oxidation via activation of AMP-activated protein kinase (AMPK), and the cross-talk of adiponectin with insulin signaling thus is thought to improve glycogen synthesis and glucose transport [149]. Plasma adiponectin levels have a negative correlation with insulin-resistance development and T2D, and adiponectin is considered as one of the strongest markers of T2D and metabolic syndrome [150]. Adiponectin and its receptors AdipoR1 and AdipoR2, known to stimulate the sensitivity of ovarian cells to insulin and gonadotropins, were detected in human follicular fluid, but also in oocytes, granulosa cells, follicular membrane cells and cumulus cells, and have been suggested to affect follicular development and ovulation [151]. Adiponectin receptors were also found in placental and endometrial cells, suggesting that this adipokine might play a crucial role in embryo implantation, trophoblast invasion and fetal growth. Adiponectin controls the steroidogenesis of ovarian granulosa cells [152] and has also been shown to decrease the production of progesterone and androstenedione in insulin-induced follicular theca cells [153].

With regard to other molecular mechanisms underlying the role of adiponectin in PCOS, granulosa cells obtained from women with PCOS were reported to express about 50% lower amounts of AdipoR1 and AdipoR2 mRNA than granulosa cells obtained from the control group (both *p* < 0.001). Furthermore, the expression of both receptors was significantly lower in obese PCOS patients than in non-obese patients [154]. Recent studies suggested that adaptor protein containing a PH domain, PTB domain and leucine zipper motif 1 (APPL1) plays a central role as the main contributing factor in adiponectin and insulin signaling. APPL1 directly interacts with the intracellular region of adiponectin receptors, thereby mediating adiponectin signaling to enhance lipid oxidation and glucose uptake. The involvement of APPL1 in insulin signaling, interacting with PI3K and AKT, suggests that it has to be considered as an important mediator of adiponectin-dependent insulin sensitization in skeletal muscles [155]. Given that, in PCOS granulosa cells, APPL1 and AdipoR1/2 were reported to be down-regulated, this interaction has to be considered to be an important molecular mechanism in PCOS pathogenesis [156].

Several studies examined adiponectin serum levels in women with PCOS. A meta-analysis of adiponectin levels in PCOS patients, including 38 trials involving 1944 PCOS women and 1654 healthy controls, showed that its levels in all women with PCOS were significantly reduced compared to the healthy controls (WMD −2.67, 95% CI: −3.22 to −2.13; *p* < 0.0001) [157]. In a meta-analysis conducted in 2020, addressing adiponectin concentrations only in lean women with or without PCOS, including 30 studies (comprising 1270 PCOS patients and 1295 controls), showed that in non-obese women, PCOS was also significantly associated with a decreased adiponectin level (SMD: −0.95; 95% CI: −1.36 to −0.53; *p* < 0.00001) [141]. Serum adiponectin levels were found to be negatively associate with the IR index in BMI-adjusted PCOS patients [158].

As previously, high-molecular-weight (HMW) adiponectin is the most abundant form in plasma and has been reported to exert the strongest metabolic effects. In maternal obesity, lower total and HMW adiponectin levels have been described in the mother, paralleled with high levels in the umbilical cord [159]. Since HMW adiponectin is particularly considered to be the best marker for IR, several studies have examined HMW levels in women with PCOS. In a recent study, HMW adiponectin was observed to be significantly lower and negatively correlated with hyperandrogenism in the PCOS group, corroborated by a study reporting an inverse correlation between HMW adiponectin and the free androgen index (FAI) [160,161]. A study including 122 women with PCOS and 89 control individuals particularly reported HMW adiponectin and the HMW/total adiponectin ratio (HMWR) to be reduced in PCOS. HMW adiponectin and HMWR were to a greater extent negatively correlated with PCOS than total adiponectin. Thus, the authors suggested HMW adiponectin and HMWR to be better markers for PCOS than total adiponectin [162].

Several interventional studies were published to increase adiponectin levels in PCOS patients. A meta-analysis conducted in 2021 revealed that metformin treatment of women with PCOS significantly increased serum adiponectin concentrations (SMD −0.58, *p* = 0.01) [163]. As another intervention to increase adiponectin levels in PCOS, omega-3 fatty acids were used, and a recent meta-analysis of 9 trials with 591 patients revealed an improvement of the HOMA index (homeostatic model assessment) of insulin resistance (WMD −0.80, *p* < 0.00001) and an increase in adiponectin levels (WMD 1.34, *p* = 0.002) [164]. These results are corroborated by the following meta-analysis conducted in 2021, comprising 5 more recent studies, including 402 women with PCOS, reporting that treatment with omega-3 fatty acids significantly increased adiponectin levels (WMD 1.42, *p* < 0.00001) in the intervention group when compared with the placebo group [165].

SNPs in the *ADIPOQ*, *ADIPOR1* or *ADIPOR2* genes, coding for adiponectin or its receptors 1 and 2, respectively, have been identified to affect metabolism and were also examined for an association with PCOS. In a recent study, the T allele and TT genotype of *ADIPOQ* SNP *rs1501299* were reported to be protective against PCOS. The GT genotype of rs1501299 increased the risk of PCOS (*p* < 0.001; OR 5.46; 95% CI 2.42–12.33) relative to the TT genotype. The GG genotype increased the risk of PCOS as well (*p* < 0.001; OR 3:00; 95% CI 1.36–6.60). Both the GT and GG genotypes of this SNP were thus reported to increase PCOS susceptibility [166]. A further study reported SNP *rs17300539* in *ADIPOQ* gene, which was previously shown to be associated with T2D, to be strongly associated with the risk of PCOS in a Chinese Han population [167]. A meta-analysis of 18 studies analyzing several *ADIPOQ* gene SNPs in women with PCOS reported that an allele of the rs1501299 polymorphism was significantly associated with PCOS risk (*p* = 0.001, OR = 1.15, 95% CI 1.06–1.26) [168]. *ADIPOQ* SNP *rs17300539,* known to affect the pathogenesis of metabolic syndrome (MS), a common comorbidity of PCOS, was reported to not be associated with PCOS with or without concomitant MS [169].

Taken together, adiponectin and particularly HMW adiponectin, being negatively correlated both with PCOS and with hyperandrogenism, are clearly suggested to beneficially affect PCOS pathophysiology by different mechanisms, such as improving IR, the regulation of hypothalamic GnRH secretion, ovarian steroidogenesis and by promoting ovulation. However, since some inconsistencies still exist regarding the connection between adiponectin and PCOS, studies, particularly further elucidating the molecular mechanisms of adiponectin actin in this disease, are necessary.

#### 2.1.4. Omentin-1 in PCOS

Omentin-1 (also referred to as intelectin-1), primarily expressed in VAT, the intestine and lungs, is also expressed in the ovaries. In women with PCOS, omentin-1 mRNA expression in granulosa cells was reported to be about four-fold increased, independently from BMI, compared to control individuals (*p* < 0.001). The same study showed about two-fold higher omentin-1 levels in FF both in obese and lean PCOS patients. Thus, omentin concentration in FF was strongly associated with omentin mRNA levels in GCs (r = 0.824, *p* < 0.001) [107]. Omentin-1 expression in human granulosa-lutein cells (hGLCs) obtained from women without PCOS was reported to be increased upon treatment with insulin, IGF-1 and metformin. It was also shown that recombinant omentin-1 enhanced IGF-1-induced progesterone and estradiol secretion in these cells and this was associated with an increase in cytochrome (CYP)19A1 protein levels and an increase in IGF-1R signaling. Furthermore, these effects were abolished when visfatin was knocked down, suggesting that omentin-1 improves IGF-1-induced steroidogenesis through the induction of visfatin in hGLCs in women without PCOS [110]. Omentin-1 levels were reported to be negatively correlated with the levels of free testosterone, and the observation of increasing testosterone levels resulting in a decrease in omentin-1 [170], suggesting the elevated free testosterone in PCOS patients to reduce levels of protective omentin-1.

A recent meta-analysis conducted on circulating omentin-1 levels, including 731 women with PCOS and 531 controls, reported a notable decrease in this adipokine in the serum of PCOS patients with an SMD of −0.67 (*p* < 0.0001), suggesting omentin-1 to play a role in PCOS [40]. Regarding non-obese women with PCOS, it was reported that plasma omentin-1 levels were also reduced in lean PCOS patients with normal glucose tolerance [170]. Both studies suggest this adipokine to be a potential PCOS biomarker. In a study including 153 non-obese PCOS patients and 114 age-matched healthy non-obese control individuals, omentin-1 serum levels were negatively correlated with HOMA-IR and fasting insulin. The authors suggested that insulin resistance could further decrease plasma omentin-1 in non-obese individuals with PCOS independent of BMI status [171]. Another study reported low omentin-1 expression in the peritoneal adipocytes of PCOS patients and demonstrated the down-regulation of omentin-1 gene expression by insulin and glucose [172], which is suggested to contribute to reduced omentin-1 levels in insulin-resistant PCOS patients. Addressing the role of omentin-1 in the inflammatory state of PCOS, lower omentin-1 levels were observed to be correlated with higher circulating TNF and lower IL-6 levels in PCOS [173]. Since IL-6 is known to act as both pro- and anti-inflammatory [174], it remains unclear whether omentin-1 contributes to the inflammatory state of PCOS.

Omentin-1 has the potential to be a good noninvasive PCOS biomarker and seems to be a promising therapeutic target in metabolic-related diseases, including PCOS, which should be further addressed in clinical studies. Additionally, further attempts are necessary to elucidate the molecular mechanisms underlying the role of this adipokine in PCOS.

#### 2.1.5. Vaspin in PCOS

Vaspin (SERPINA12) is another adipokine protecting from inflammation and insulin resistance. Studies on the relation between vaspin levels and obesity achieved conflicting results. A meta-analysis conducted on this topic, including 1826 participants, showed that vaspin’s serum level was 0.52 ng/mL higher (*p* = 0.02) in obese than in non-obese individuals. For the analysis of vaspin levels in patients with T2D, 11 studies using 1570 patients were included and resulted in the observation of an even more significant increase in vaspin levels in T2D patients than in the control group (*p* < 0.00001) [175]. A previous study observed that the levels of this adipokine reached the highest values at the disease phase when plasma insulin levels and obesity peaked, whereas, with progression of T2D, levels of vaspin decreased. The same study reported vaspin to exert an insulin-sensitizing effect on WATs in states of obesity [176].

Serum vaspin level has been reported to be increased in women with PCOS. The latest meta-analysis of 88 studies reported the serum levels of vaspin to be significantly elevated in women with PCOS (SMD 0.69; *p* = 0.004) [106]. Supporting these findings, a previous study showed that vaspin mRNA and protein expression in adipose tissue were notably increased in PCOS patients and were induced by glucose. On the other hand, treatment with metformin was observed to decrease vaspin plasma levels in PCOS patients [177]. Vaspin was shown to be highly expressed in the human ovary and to stimulate granulosa cell steroidogenesis, proliferation and viability in a concentration-dependent manner through receptor GRP78 (*p* < 0.0001). The authors concluded that vaspin appears as a novel modulator of human granulosa cell physiology and suggested it might play a protective role in PCOS pathophysiology [178]. Genetic polymorphisms (SNPs) in the vaspin gene *SERPINA12* were identified, which strongly correlated with vaspin serum levels with *p*-values up to 10^−35^, but no association with T2D or related traits were observed [179]. With regard to PCOS, in a very recent study including 40 women with PCOS and the same number of control individuals, the C allele of SNP C677T in the vaspin gene was reported to increase the risk of PCOS 2.077 times (*p* < 0.05), and thus was suggested to be a genetic susceptibility marker for PCOS [180]. In a previous study including 150 women with PCOS and the same number of controls, the vaspin gene SNP rs2236242 exhibited significant differences in genotype frequencies between both groups (OR = 0.59, CI = 0.37–0.95, *p* = 0.03). The A allele decreased the risk of PCOS (OR = 0.67, CI = 0.46–0.96, *p*=0.03) compared to the T allele. Since there was no significant association between vaspin rs2236242 gene polymorphism and PCOS after adjusting genotypes for BMI, the authors suggested this association was affected by obesity status [181]. With regard to the epigenetic variations of the vaspin gene, at present, no data have been published.

Taken together, the anti-inflammatory action of vaspin, its ability to counteract IR and its ability to activate GC proliferation and viability, thus maintaining ovarian granulosa cell function, suggests that this adipokine may act as a protective factor for PCOS. However, given that not all studies on this topic are consistent, more basic and interventional studies further elucidating the role of vaspin in PCOS are necessary.

#### 2.1.6. Apelin in PCOS

Apelin, which is elevated in the serum of obese or diabetic individuals, has been reported to protect from hypertension, glucose intolerance and insulin resistance [25]. Different experimental strategies have led to the identification of apelin as adipokine also regulating the HPG axis, ovarian angiogenesis and follicle development [182]. Apelin receptor APLNR has been detected in hypothalamic nuclei and in the anterior pituitary, and thus might be involved in the control of reproduction [183]. Apelin and its receptor APLNR are also expressed in granulosa cells and in cumulus and theca cells. In the normal ovary, apelin has been reported to increase granulosa cell proliferation and to decrease apoptosis [184]. Furthermore, apelin induced basal, FSH- and IGF-1-induced steroidogenesis [185]. Mice injected with apelin-13 exhibited decreased serum levels of testosterone, LH and FSH, hindering their reproductive function. Progesterone has been reported to induce ovarian APLNR expression [186]. In cultured granulose cells obtained from nonobese patients without PCOS, IGF-1 increased APLNR expression, and recombinant human apelins-13 and -17 increased both basal and IGF-1-induced steroid secretion [187]. In patients with PCOS, FF apelin levels and granulose cell apelin and APLNR mRNA expression were reported to be higher than in the control patients [187]. Elevated apelin and APLNR protein and mRNA levels in the FF and granulosa cells of PCOS patients were also observed in a later study [107]. Whereas apelin levels in FF were elevated in both obese and lean women with PCOS, apelin and APLNR expression in granulose cells were only increased in obese PCOS patients. Apelin and vascular endothelial growth factor (VEGF) expression in the granulose cells of PCOS patients was activated by hyperinsulinemia [186]. Thus, in women with PCOS, apelin is suggested to exert beneficial actions by supporting angiogenesis in ovarian follicles via VEGF, and by improving ovarian steroidogenesis via IGF-1 [188,189].

The search for a change in serum apelin levels in women with PCOS achieved inconclusive results. However, the latest meta-analysis conducted in this regard, including 88 case-control studies, observed no statistically significant difference of circulating apelin levels between women with and without PCOS [106], being supported by another recent study including data obtained from 81 studies comparing non-obese PCOS patients and controls [141]. Thus, apelin serum levels do not seem to be associated with the pathogenesis of PCOS. Interestingly, apelin concentrations in FF have been reported to be higher than in plasma, suggesting that follicular apelin at least in part originates from granulosa cell secretion and regulates the function of granulosa cells in a paracrine and/or autocrine manner, as described above [182,190].

In conclusion, apelin has been suggested to improve glucose tolerance and insulin sensitivity, thus counteracting IR often being associated with PCOS. Its elevated concentration in FF is suggested to increase the IGF-1-induced secretion of estrogens and progesterone as well as the expression of VEGF in granulose cells. Many studies suggested apelin to be involved in follicular development and that this adipokine might affect cyst formation and ovulatory dysfunction in PCOS patients. The mostly positive actions of apelin to date suggest a beneficial effect of this adipokine in women with PCOS.

#### 2.1.7. Follistatin in PCOS

Follistatin was initially identified as a component of follicular fluid capable of inhibiting the follicle-stimulating hormone (FSH) [191]. Later, follistatin expression was shown in a variety of tissues, including ovarian, pituitary and WAT. Follistatin has been recognized as a high-affinity binding and neutralizing protein for several members of the TGF-β superfamily, including activins and MST1 (macrophage stimulating 1) [192]. Given that the TGF-β–MST1 axis is known to suppress the development of BAT, the inhibition of this axis by follistatin was demonstrated to lead to WAT browning, mitochondrial biogenesis and thereby the activation of BAT thermogenesis [193]. The activation of BAT in rats with PCOS by cold exposure reduced the circulating levels of testosterone and LH and decreased ovarian expression of steroidogenic enzymes, leading to improved ovulation and fertility [194]. Thus, follistatin has been suggested to have therapeutic potential for the treatment of metabolic disorders and PCOS through the increase in BAT mass and activity [193]. BAT activation has also been reported with regard to the related adipokine follistatin-like 1 (FSTL1), as FSTL1 is also a potent inhibitor of TGF-β superfamily proteins [195]. FSTL1 haploinsufficiency in mice led to reduced thermogenic gene expression, impaired BAT recruitment and decreased heat production. However, FSTL1 is able to use additional pathways to activate BAT. FSTL1 promoted β3-adrenergic signaling, which was shown to be required to up-regulate PPARγ and UCP1 to activate thermogenesis in brown adipocytes [196].

A recent meta-analysis including 9 studies with 815 PCOS cases and 328 controls reported significantly increased circulating follistatin levels in PCOS patients compared with the control group (WMD = 0.44 ng/mL; 95% CI = 0.30–0.58, *p* < 0.001). Follistatin serum levels were also significantly higher in non-obese women with PCOS than in non-obese controls, and in obese PCOS patients compared to obese-control individuals, but no significant difference in follistatin levels was observed in obese PCOS patients compared to non-obese women with PCOS [79]. Thus, the fact that circulating follistatin levels were elevated in women with PCOS, independent of BMI, further supported the proposed contribution of this adipokine to the pathology of PCOS. Additionally, follistatin was suggested to be a useful biomarker indicating the course of this disease, e.g., for the treatment of women with PCOS. In contrast to follistatin, levels of circulating FSTL1 protein were elevated in obese women. FSTL1 also was reported to be associated with insulin resistance and T2D [197].

In conclusion, follistatin and FSTL1 trigger the development of BAT, leading to an increase in mitochondrial biogenesis and to the activation of BAT thermogenesis. As we determined later in the paper, BAT was reported to be able to reverse crucial features of PCOS, resulting in improved ovulation and fertility with a reduction in cystic ovarian follicles. Follistatin treatment therefore might be a therapy option for women with PCOS. Since follistatin serum levels were elevated in PCOS patients in an obesity-independent manner, it might also have the potential as a non-invasive biomarker for PCOS.

#### 2.1.8. Visfatin in PCOS

Visfatin is the extracellular form of nicotinamide phosphoribosyltransferase (NAMPT). Visfatin secreted from WAT, elevated in the serum of individuals with T2D, metabolic syndrome or obesity, was shown to exert inflammatory and profibrotic activities [70,71,72]. Visfatin is suggested so contribute to adipose tissue fibrosis being related to insulin resistance, liver steatosis and metabolic diseases [74]. Human granulosa cells, cumulus cells, oocytes and follicular membrane cells express and secrete visfatin [198], and an autocrine loop might be responsible for visfatin-induced GC proliferation. The number of oocytes retrieved from women under controlled ovarian stimulation increased with elevating levels of visfatin in follicular fluid, suggesting a beneficial effect of this adipokine on female reproduction [199]. In bovines, visfatin was reported to activate GC steroidogenesis increasing the secretion of P4 and E2, proliferation and oocyte maturation [200,201].

The latest studies support the adverse effects of visfatin on PCOS. Using a hyperandrogenic PCOS mice model exhibiting elevated visfatin levels, the inhibition of visfatin by a specific antagonist significantly reduced androgen and testosterone serum levels, suppressed cyst formation, promoted corpus luteum formation and increased ovarian glucose content [202]. Thus, the authors suggested that visfatin inhibition could have a therapeutic potential in PCOS management along with other interventions.

There have been contradictory reports on the serum levels of visfatin in women with PCOS. However, the results of the most recent studies tend towards elevated visfatin levels in women with PCOS. A recent case-control study including 140 non-obese PCOS patients and the same number of BMI-matched controls [203] observed higher serum visfatin levels in the PCOS group than in the healthy controls, but even more elevated visfatin levels in hyperandrogenic PCOS versus non-hyperandrogenic PCOS women. Visfatin levels showed a strong positive correlation with insulin resistance, followed by the free androgen index (FAI) in PCOS cases irrespective of BMI, suggesting an adverse effect of visfatin on PCOS pathophysiology among non-obese women. Another recent study also reported elevated visfatin serum levels in PCOS patients compared to the control group, also reporting ovarian visfatin expression to be associated with its serum levels, with levels of fasting serum insulin, LH, testosterone, free androgens and with HOMA-IR and the LH/FSH ratio [204].

Taken together, the role of visfatin in PCOS remains inconclusive, which might result from the different experimental approaches used in the previously mentioned studies. However, further studies are needed to elucidate its actions in PCOS and the underlying molecular mechanisms.

#### 2.1.9. Brown Adipose Tissue and Batokines in PCOS

Brown adipose tissue (BAT) is activated, e.g., by cold exposure via noradrenaline released by sympathetic nervous activities [205]. BAT plays a key role in metabolism and energy expenditure through adaptive thermogenesis, which is mediated by the high number of mitochondria in BAT cells. BAT activity has been suggested to be associated with protection from obesity and metabolic diseases like dyslipidemia and T2D [206]. Notably, the transplantation of BAT into the visceral cavity of mice improved their glucose tolerance, increased insulin sensitivity, lowered body weight and decreased fat mass [207]. BAT secretes a higher number of so-called brown adipokines, regulatory factors exerting various endocrine functions and also regulates BAT differentiation and activity [18]. Some brown adipokines have hormonal functions improving the metabolic profile of glucose and lipid homeostasis. Additionally, they are responsible for the browning of WAT [207,208].

Decreased activity of BAT was observed both in human PCOS patients and in a PCOS rat model [209]. Dysfunction of adipose tissue promotes metabolic disorders in the peripheral tissues of PCOS patients with lower activity of lipoprotein lipolytic enzyme. The activation of BAT has been suggested to be a potential therapeutic option for the treatment of women with PCOS as it exerts beneficial effects on IR [210] and obesity [211]. Cold exposure and stimulation with specific drugs were reported to have the same effect as noradrenaline on induction of WAT browning, leading to the development of beige adipocytes with similar functions as brown adipocytes in murine SAT [212]. In rat PCOS models, the whitening of BAT can be observed. In this model, BAT whitening could be reversed by the cold exposure of rats with PCOS (4 °C for 20 days), and the batokines secreted by the reactivated BAT normalized the estrous cycle, decreased the serum levels of testosterone, LH and inflammatory factors and led to improved ovulation [194]. Notably, transplantation of BAT was reported to reverse polycystic ovaries, and improve infertility and IR in rodents with PCOS [111]. BAT transplantation was also reported to enhance endogenous BAT activity and to increase the level of circulating adiponectin and insulin sensitivity, thereby positively affecting acyclic polycystic ovaries, hyperandrogenism and infertility of rats with PCOS [213].

Since neither cold treatment nor BAT transplantation seem to be an option for the treatment of women with PCOS in the clinical situation, various efforts have been made to identify natural compounds being able to activate BAT in patients with PCOS. Recently, a promising substance activating BAT, the flavonoid rutin, was identified, which was able to improve insulin sensitivity in rats with PCOS and to upregulate the expression of ovarian steroidogenic enzymes, resulting in the observation of a reduced cyst formation and an elevated number of mature follicles [214]. Ongoing studies to obtain compounds able to activate the BAT of human PCOS patients are underway.

The effects of BAT on PCOS, as described above, are mediated by the secretion of a set of adipokines called brown adipokines (also referred to as batokines). FGF21, IL-6, neuregulin 4, VEGF-A and bone morphogenetic protein 8b (BMP8b) were among the first BAT-derived endocrine factors to be identified [18,206]. Other adipokines, such as chemerin and adiponectin, previously known as WAT adipokines, have recently been identified to also be secreted from BAT [18]. The progress of proteomics technology allowed the identification of a large set of batokines; however, in three proteomic studies examining the secretome of cultured, thermogenically stimulated BAT cells in response to noradrenaline [43,44] or cAMP [42], a Venn diagram analysis revealed five batokines in the overlap of all mentioned studies, namely, two ECM components, collagen α-1 (III) chain (COL3A1) and procollagen C-proteinase-enhancer protein (PCOLCE), and additionally insulin-like growth-factor-binding protein-4 (IGFBP4), follistatin-like protein-1 (FSTL1) and chemerin [18]. Since the roles of chemerin and FSTL1 in PCOS have already been addressed in the context of WAT-secreted adipokines, the role of a previously mentioned classical and newly identified batokine in PCOS is later summarized.

##### Batokine Adiponectin as Mediator of BAT Effects on PCOS

As mentioned above, adiponectin is a very potent insulin sensitizer, protecting from type-2 diabetes, and exhibits decreased serum levels in obesity and PCOS [29]. In addition to the results summarized in Section 2.1.3, since adiponectin was identified to be released from BAT as brown adipokine [18,215], its role as a possible mediator of the beneficial effects of BAT on PCOS pathophysiology was examined in recent studies. BAT activity has been reported to be significantly reduced in women with PCOS [209]. BAT transplantation into genetically obese mice reversed obesity, activated endogenous BAT and increased circulating adiponectin levels [216]. In a subsequent animal study, the transplantation of BAT into a PCOS rat model activated the reduced BAT activity, and after 3 weeks could partially reverse the PCOS phenotype, including cystic follicles, leading to the normalization of ovarian steroidogenesis, and was able to reverse glucose intolerance, insulin resistance, LH levels, the FSH/LH ratio, acyclicity and infertility [213]. Notably, the treatment of PCOS rats (w/o transplanted BAT and exhibiting decreased adiponectin levels) with recombinant adiponectin for 20 days was able toat least in partrecapitulate the beneficial effects of BAT transplantation in the PCOS rats [213]. These data suggest BAT-secreted adiponectin might be a mediator of the beneficial effects of BAT transplantation on the pathobiology of PCOS rats.

##### FGF21 in PCOS

Fibroblast growth factor (FGF) 21 was one of the first endocrine factors identified to be secreted by BAT and is thus considered as a batokine. FGF21 has been shown to be a cold-induced endocrine activator of the BAT function in humans [208]. In adipose tissue, FGF21 promotes glucose utilization and increases energy expenditure by enhancing adipose tissue insulin sensitivity and BAT thermogenesis. Therefore, FGF21 favors glucose consumption for heat production in BAT instead of energy storage. In addition to its endocrine action in BAT, cold exposure stimulates the local production of FGF21 in brown adipocytes. FGF21 also induces the browning of white adipocytes and generation of dispersed brown-like adipocytes in white adipose depots [217]. Being predominantly expressed in the liver, it is an important metabolic regulator that controls energy homeostasis. As FGF21 treatment in animal obesity models resulted in reduction of adiposity, improved insulin sensitivity, a decrease of cholesterol and triglyceride levels, FGF21 was suggested to have potential as a novel therapy for obesity and related diseases like T2D and PCOS. Endogenous FGF21 serum levels, however, are increased during obesity-related diseases, suggesting the development of FGF21 resistance during obesity and the successive lowering of FGF21 efficacy.

Studies addressing circulating FGF21 levels in women with PCOS achieved contradictory results. A recent study reported FGF21 serum levels to be significantly increased in PCOS patients compared to women without PCOS, suggesting the possible involvement of FGF21 in PCOS [218], supported by a previous study reporting elevated FGF21 levels in PCOS patients, which were associated with levels of testosterone, LH and with BMI [219]. However, a conflicting study observed higher FGF21 levels in obese women without PCOS than in obese and non-obese PCOS patients, and elevated FGF21 serum levels in obese PCOS patients compared to non-obese PCOS patients. Circulating FGF21 levels were correlated with BMI and insulin resistance (HOMA-IR), but not with serum levels of estrogens or free androgens, demonstrating FGF21 not to be associated with PCOS, but with obesity and IR [220].

Taken together, the results obtained from animal studies showing FGF21 not only to increase insulin sensitivity and to reduce adiposity, but also to promote BAT development and function, which has been shown to substantially improve the polycystic ovarian phenotype, IR and infertility in animal PCOS models, suggest an important beneficial effect of FGF21 on PCOS pathophysiology. However, further studies are needed to examine the extent to which these effects are also present in humans and to clarify the inconclusive reports on circulating FGF12 in women with PCOS.

**Figure 1:**
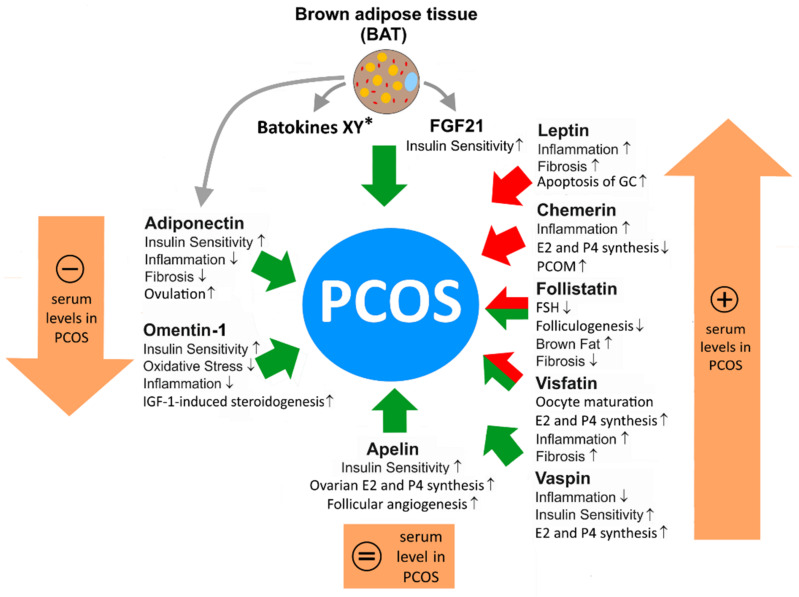
Proposed effects of the indicated WAT adipokines and of BAT on PCOS, based on current experimental evidence and on an assessment of the effects of the known adipokine actions on PCOS. Green arrows illustrate a beneficial effect, red arrows an adverse effect on PCOS pathophysiology. Red-green-colored arrows indicate the presence of both beneficial and adverse effects of an adipokine. BAT activation or transplantation has been reported to notably improve PCOS, although only few molecular mechanisms underlying this effect are known to date. * Novel proteomic approaches are expected to identify further batokines (XY) affecting PCOS. “↑” = increase; “↓” = decrease.

In conclusion, adipokines are able to support or counteract the pathobiology of PCOS by the various mechanisms discussed above (Figure 1). Whereas the action of certain adipokines seems to be sufficiently understood to examine whether the application or inhibition of these molecules might be a therapeutic option for the treatment of PCOS in animal models or later in clinical studies, there is a variety of other adipokines and batokines identified by current proteomic analyses of the WAT and BAT secretome, which might have potential as future drugs for the therapy of PCOS.

## 3. Endometriosis

Endometriosis is a disorder defined by the presence of endometrium-like tissue in any extrauterine site [221]. The prevalence is around 2–10% in women of a reproductive age; however, the estimated number of unreported cases is higher. First described in 1690, many theories tried to explain its pathogenesis. One of the key hypotheses for the pathogenesis of endometriosis is Sampson’s retrograde menstruation theory [222]. As underlying mechanisms, physical factors, such as uterine tissue damage or scarring (e.g., during surgery), the uterine microenvironment, stem cells, remnant cells from menstrual blood, and biochemical and genetic factors are considered, like hormones and other gene products regulating inflammation, apoptosis, invasion, angiogenesis, autophagy and oxidative stress [221,223,224]. Based on their location, three types of endometriosis have been characterized: ovarian endometriomas, superficial peritoneal endometriosis, and deep endometriotic nodules [221,225]. Although being a benign disease, some similarities with cancer exist regarding invasion and metastasis. Thus, expression of various tumor-promoting genes coding for cell cycle regulators, metalloproteinases and angiogenic proteins is upregulated in endometriosis, whereas genes of the apoptotic machinery are downregulated.

One of the main mechanisms for the initiation of endometriosis is chronic inflammation [226]. Macrophages, natural killer cells, T cells and dendritic cells are regulated by proinflammatory mediators, such as cytokines, prostaglandins and chemokines [227]. These immunoactive molecules promote adhesion and support the evasion of immunosurveillance, being major mechanisms for the adhesion of endometrial cells in ectopic sites. Moreover, chronic inflammation is also responsible for endometriosis-associated symptoms, such as infertility and pain [221,228].

As endometriosis is a hormonal-regulated disease, the impact of estrogens and estrogen receptors (ERs) on pathogenesis is evident. An imbalance in estrogen and progesterone levels leads to an impaired proliferation of the endometrium, as well as insufficient the decidualization of the human uterus. In endometriosis, the efficiency of progesterone and estrogens is altered, which leads to progesterone resistance and an excess of estrogens [229,230]. Due to the increased influence of estrogens, the local infiltration of immune cells and inflammation is triggered. Moreover, ERs and progesterone receptors (PRs) play a relevant role in the proliferation and differentiation of normal endometrium; both ERα and ERβ are known to be crucial factors to maintain the physiological functions of the endometrium. ERβ in particular targets several pathways regulating prostaglandin synthesis, which is associated with the induction of inflammation and prevention of apoptosis [228,230]. Moreover, various tumor-promoting and angiogenic proteins are activated by ERα, and an epithelial–mesenchymal transition (EMT) is induced, leading to the progression of endometrial lesions [230].

Taken together, endometriosis is a multifactorial disorder and the main molecular mechanisms underlying its pathogenesis are activation of estrogen signaling, tumor-promoting angiogenic and invasion-associated proteins, inflammatory molecules and the loss of apoptotic proteins. In addition to hormone imbalance and oxidative stress, these dysregulated pathways are primarily responsible for the metastasis of endometrial cells into extrauterine tissues.

### 3.1. BMI and Endometriosis

To date, there is convincing evidence for an inverse association of endometriosis and BMI [231]. Low overall adiposity and a preferred localization of fat below the waist were found to be related to endometriosis [232]. The risk of endometriosis is higher for females being thinner than average at an age of about 10 years [233]. In later life, abdominal fat distribution contributes to the development of endometriosis [233]. Overweight/obesity is a risk factor for various diseases, and obesity as well as endometriosis are associated with low-grade inflammation and higher estrogens, and thus an inverse relation is exceptional [231]. Associations of endometriosis with body-fat distribution and metabolic diseases, such as insulin resistance, have not been resolved. It is, however, relatively clear that obesity does not protect women from endometriosis. The lower BMI of patients with endometriosis was also supposed to be a consequence rather than cause of the disease. Chronic pain can decrease appetite, and this may account for weight loss [231]. MicroRNAs differentially expressed in patients with endometriosis were shown to lower adipocyte stem cell numbers and this may cause a loss of fat mass [234]. Fat-tissue-derived mesenchymal stem cells ameliorated endometriosis-related inflammation in an experimental model, suggesting that fat mass loss contributes to disease progression [235].

Thus, the inverse relation of BMI and endometriosis suggests the role of adipokines in this common disease. Adipose tissue in the peritoneal cavity was supposed to contribute to the pathogenesis of endometriosis, and the role of locally produced adipokines has been demonstrated in several studies [231,236].

### 3.2. Role of Adipokines in Endometriosis

#### 3.2.1. Role of Leptin in Endometriosis

Leptin is the most studied adipokine in endometriosis. Serum leptin is positively correlated with BMI and body fat [22]. Higher serum leptin levels were observed in women with endometriosis in comparison to the controls. Here, serum leptin positively correlated with BMI in both cohorts [237]. A meta-analysis published in 2021 also showed higher leptin levels in the serum of women with endometriosis [35]. This analysis also observed that leptin declined in patients with advanced-stage disease in comparison to patients with early endometriosis. A case-control study within the prospective Nurses’ Health study had access to the blood of 29.611 females. During the study, 350 women developed endometriosis; however, an association with leptin levels was not observed [238].

Two recent meta-analyses also came to the conclusion that serum and plasma leptin levels did not differ between patients with endometriosis and the respective controls [239,240].

Omental fat was shown to express leptin [241], and thus peritoneal leptin levels were also measured. Here, it must be noted that leptin concentrations in peritoneal fluid are about two-to-four-fold lower than its serum levels. Peritoneal leptin was indeed higher in patients and positively correlated with BMI in the patient and control cohorts [237]. Two meta-analysis provided further evidence for higher leptin concentrations in the peritoneal fluid of women with endometriosis compared to the respective controls [239,240].

Endometrial tissues of 44 women with endometriosis were further shown to express higher levels of leptin and leptin-receptor protein than tissues obtained from 42 non-affected women [242]. The expression of these proteins was not linked to disease severity. Endometrial leptin mRNA expression, however, did not differ between patients and controls [243]. It was also suggested that microRNAs, which differ in the serum of patients with endometriosis and controls, altered the leptin levels released by fat tissues [234].

Experimental studies reported leptin to significantly enhance the proliferation of both eutopic and ectopic endometrial stromal cells in endometriosis [244]. Furthermore, leptin was shown to stimulate the migration and invasion of endometrial cells. The binding of leptin to its receptor and activation of the Janus kinase 2/signal transducer and activator of transcription-3 (JAK2/STAT3) signaling pathway caused the up-regulation of matrix metalloproteinase-2 (MMP-2), leading to increased cell invasion and migration [245]. Furthermore, leptin has been shown to be essential for angiogenesis in an endometriosis mouse model, and studies reporting leptin to activate VEGF-A expression and the detection of elevated VEGF-A in endometriosis both suggest that the angiogenic effect of leptin is mediated via this growth factor [246,247,248].

**Figure 2:**
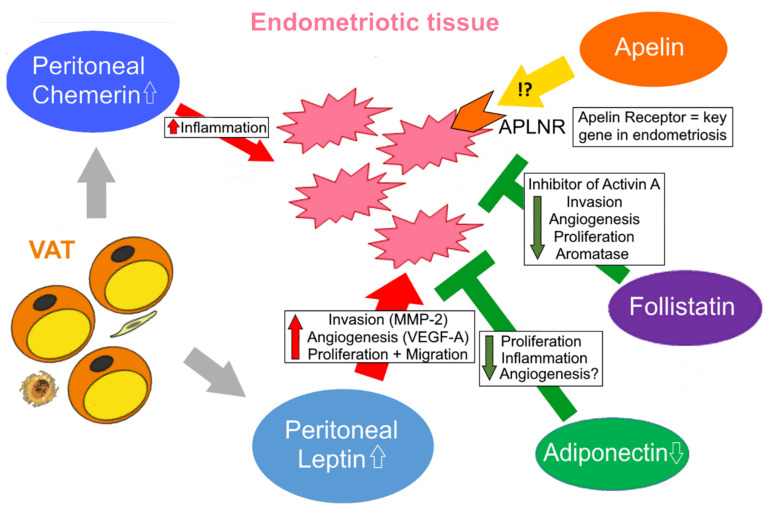
Proposed effect of the indicated adipokines on endometriotic tissue based on the current state of research. Additional studies are necessary to further substantiate the role of these adipokines in endometriosis. It can be expected that the recent report suggesting apelin receptor APLNR to be one of three key genes in endometriosis resulting from in silico analyses of DNA microarray data will encourage functional studies to elucidate underlying molecular mechanisms. Red arrows indicate adverse effects on endometriosis; green inhibitor arrows indicate beneficial effects. In text boxes, red arrows indicate the up-regulation and green ones the down-regulation of the respective pathways. White arrows indicate an increase or decline in the adipokine concentration in the blood/peritoneal fluid of women with endometriosis.

Although the association of leptin with disease severity is controversial, there is convincing evidence that leptin primarily exerts adverse effects on endometriotic tissue via the activation of proliferation, invasion, migration and neo-angiogenesis (Figure 2).

#### 3.2.2. Role of Adiponectin in Endometriosis

In an endometriotic cell line, recombinant leptin was shown to up-regulate adiponectin mRNA levels [242]. There is also evidence that microRNAs differentially abundant in endometriosis serum affect adipocyte function and adiponectin release [234]. Adiponectin is an anti-inflammatory adipokine, and moreover regulates angiogenesis and cell proliferation, which are pathogenic factors in this disease. Recombinant adiponectin inhibited the cell proliferation of primary human endometriotic stromal cells, and this effect was achieved by adiponectin concentrations that were much lower than the serum levels [249]. Adiponectin was also shown to reduce the viability of normal endometrial stromal cells [250].

Adiponectin- and adiponectin-receptor protein levels were similarly abundant in endometrial tissues of women with endometriosis, and the controls and did not correlate with the disease state [242].

In contrast, it was also reported that peritoneal fluid adiponectin was lower in patients with endometriosis in comparison to the controls [251]. Patients with stage-III/IV endometriosis had less peritoneal fluid adiponectin levels than stage-I/II patients. Serum adiponectin also negatively correlated with the endometriosis stages in a different cohort, and was lower in the serum of women with endometriosis compared to the controls [252].

Though an association of peritoneal and circulating adiponectin with disease stages was not identified in a recent meta-analysis, a decline in the adiponectin concentration was noticed in the endometriosis patients [35].

In conclusion, adiponectin is known to exert multiple beneficial, health-protective effects, e.g., via its anti-inflammatory action, and may also limit endometriosis, as was suggested by the inhibition of proliferation. Additional studies are required to further substantiate the effects of adiponectin on this disease.

#### 3.2.3. Role of Chemerin in Endometriosis

Chemerin is a chemoattractant for immune cells and might provide a link between endometriosis and inflammation [253]. Ovarian endometrioma tissue expressed higher levels of chemerin and CMKLR1 mRNA and protein than the eutopic endometrium of controls. The 31 women with endometriosis had similar serum chemerin as the 48 controls. In peritoneal fluid, chemerin was, however, induced. A positive correlation of chemerin with IL-6 and TNF, which were both higher in patients, was observed [254]. To the best of our knowledge, there are only two studies that analyzed the role of chemerin in endometriosis. A higher abundance of chemerin and its receptor in the diseased state shows that further research is needed on the role of chemerin in endometriosis.

#### 3.2.4. Role of Follistatin in Endometriosis

Activin A regulates inflammation and cell proliferation and is abundant in endometrial proliferative disorders. There is convincing experimental evidence that activin A promotes the occurrence of endometriosis [255]. Recombinant activin A was shown to stimulate aromatase expression and to enhance fibrosis in endometrial stromal cells, and to increase the invasion of endometrial stromal and epithelial cells into a modeled peritoneum [256]. Thus, current experimental data suggest that a dysregulation of activin A and follistatin, a natural inhibitor of activin A bioactivity, may contribute to endometriosis.

Furthermore, it was shown that the biological effects of activin A regarding the regulation of IL-6, IL-8 and VEGF differed between the human endometrial stromal cells obtained from women with endometriosis and controls. Importantly, follistatin was an effective antagonist of activin A in the cells of patients and controls, demonstrating the patients’ cells to be still sensitive to the antagonistic effects of follistatin [257].

Inflammatory cytokines, such as TNF and IL-1 beta, induced activin A and also increased follistatin in endometrioma stromal cells. Follistatin mRNA and protein were accordingly more abundant in ovarian compared to healthy endometriotic tissues [258].

Women with ovarian endometrioma (52 patients) had increased serum follistatin than patients with other benign ovarian cysts (52 patients), patients with non-ovarian endometriosis (11 patients) and healthy controls (27 controls). Notably, women with non-ovarian endometriosis had higher serum follistatin levels than patients with other benign ovarian cysts and healthy controls. A cut-off value of 1025 pg/mL had a 100% sensitivity and a 96% specificity for the detection of endometrioma among normal controls. A cut-off value of 1433 pg/mL detected patients with a 92% sensitivity and 92% specificity among all women with ovarian cysts [259].

Taken together, the role of follistatin as particularly an inhibitor of activin A confers a beneficial effect of this adipokine on endometriosis.

#### 3.2.5. Apelin in Endometriosis

Notably, a recent bioinformatical analysis of microarray expression data identified the apelin receptor APLNR as one of three key genes in endometriosis [260]. Gene-set-enrichment analysis revealed that focal adhesion and extracellular matrix receptors are enriched in the group with high expression. Although, to date, there are no further studies to elucidate the role of the apelin receptor in endometriosis, studies addressing this fact can be expected.

In conclusion, adipokines affect the pathophysiology both of PCOS and endometriosis. However, the role of adipokines in PCOS to date is more appropriately examined. The increasing capabilities of multi-omics approaches are strongly expected to shed a brighter light on the role of adipokines in both diseases.

## Figures and Tables

**Table 1:** Primary functions of the indicated adipokines and their serum levels in obesity.

Adipokine	Serum Levels in Obese Individuals	Primary Functions
Leptin	+	Suppresses food uptake and regulates energy homeostasis, induces fibrosis
Adiponectin	−	Promoter of insulin sensitivity + hepatocyte fatty acid oxidation, reduces gluconeogensis + inflammation
Omentin-1	−	Reduction in oxidative stress, inflammation + apoptosis
Chemerin	+	Leukocyte chemotattractant, involved in regulation of insulin response, blood pressure and adipogenesis
Vaspin	+	Anti-inflammatory promoter of glucose deposition and of insulin sensitivity
Apelin	+	Promoter of glucose tolerance, insulin sensitivity, fatty acid oxidation and of mitochondrial biogenesis
Visfatin	+	Activation of inflammatory pathways, promoter of vascular remodeling and fibrogenesis
Follistatin	=	Antagonist of members of the TGF-β superfamily, including activins, and protects from metabolic disease

“+” = elevated levels; “−“ = decreased levels; “=” = unchanged levels.

**Table 2:** Adipokines and their main effects in women with PCOS.

Adipokine	Serum Levels in PCOS	Main Effects in PCOS
Leptin	+	Insulin sensitivity ↓, inflammation ↑, fibrosis ↑, GnRH secretion ↑, P4 synthesis ↑, apoptosis ↑
Chemerin	+	Insulin sensitivity ↓, ovarian E2 and P4 synthesis ↓, inflammation ↑, PCOM ↑
Adiponectin	−	Insulin sensitivity ↑, ovulation ↑, inflammation ↓, fibrosis ↓
Omentin-1	−	Insulin sensitivity ↑, inflammation ↓, IGF-1-induced steroidogenesis in GC ↑
Vaspin	+	Insulin sensitivity ↑, inflammation ↓, GC viability and steroidogenesis ↑
Apelin	=	Insulin sensitivity ↑, ovarian E2 and P4 synthesis ↑, follicular angiogenesis ↑
Follistatin	+	Activin-triggered FSH release ↓, folliculogenesis ↓, fibrosis ↓
Visfatin	+	Oocyte maturation ↑, androgen level ↓, ovarian E2 and P4 synthesis ↑, inflammation ↑

E2: 17-β estradiol; FSH: follicle-stimulating hormone; GC: granulosa cell; GnRH: gonadotropin-releasing hormone; IGF-1: insulin-like growth factor 1; P4: progesterone; PCOM: polycystic ovary morphology. “+” = elevated levels; “−“ = decreased levels; “=” = unchanged levels; “↑” = increase; “↓” = decrease.

## Data Availability

Not applicable.
